# *Escherichia coli* K-12 Survives Anaerobic Exposure at pH 2 without RpoS, Gad, or Hydrogenases, but Shows Sensitivity to Autoclaved Broth Products

**DOI:** 10.1371/journal.pone.0056796

**Published:** 2013-03-08

**Authors:** Daniel P. Riggins, Maria J. Narvaez, Keith A. Martinez, Mark M. Harden, Joan L. Slonczewski

**Affiliations:** Department of Biology, Kenyon College, Gambier, Ohio, United States of America; National Institutes of Health, United States of America

## Abstract

*Escherichia coli* and other enteric bacteria survive exposure to extreme acid (pH 2 or lower) in gastric fluid. Aerated cultures survive via regulons expressing glutamate decarboxylase (Gad, activated by RpoS), cyclopropane fatty acid synthase (Cfa) and others. But extreme-acid survival is rarely tested under low oxygen, a condition found in the stomach and the intestinal tract. We observed survival of *E. coli* K-12 W3110 at pH 1.2–pH 2.0, conducting all manipulations (overnight culture at pH 5.5, extreme-acid exposure, dilution and plating) in a glove box excluding oxygen (10% H_2_, 5% CO_2_, balance N_2_). With dissolved O_2_ concentrations maintained below 6 µM, survival at pH 2 required Cfa but did not require GadC, RpoS, or hydrogenases. Extreme-acid survival in broth (containing tryptone and yeast extract) was diminished in media that had been autoclaved compared to media that had been filtered. The effect of autoclaved media on extreme-acid survival was most pronounced when oxygen was excluded. Exposure to H_2_O_2_ during extreme-acid treatment increased the death rate slightly for W3110 and to a greater extent for the *rpoS* deletion strain. Survival at pH 2 was increased in strains lacking the anaerobic regulator *fnr*. During anaerobic growth at pH 5.5, strains deleted for *fnr* showed enhanced transcription of acid-survival genes *gadB*, *cfa*, and *hdeA*, as well as catalase (*katE*). We show that *E. coli* cultured under oxygen exclusion (<6 µM O_2_) requires mechanisms different from those of aerated cultures. Extreme acid survival is more sensitive to autoclave products under oxygen exclusion.

## Introduction

Extreme-acid resistance (or acid survival) is defined as the ability of neutralophilic bacteria such as *Escherichia coli* to survive at pH levels too acidic to permit growth; for *E. coli* K-12, this is typically pH 2 [1]–[3]. In aerated cultures of *E. coli*, acid resistance involves numerous acid response systems such as the amino acid-dependent glutamate and arginine decarboxylases [4]–[6]. Most of these acid resistance systems are up-regulated during growth in moderate acid (pH 5.5) and require specific media components and conditions [1], [7]–[10] Acid-stress regulons include oxidative stress regulators such as *rpoS*, which activates the Gad acid resistance regulon[4], [8].

Acid survival is rarely tested under conditions excluding oxygen [9], [11]. Noguchi *et al.* (2010) show a contribution of hydrogenases, particularly hydrogenase-3, for extreme-acid survival using sealed screw-cap tubes, but the assays involve dilution and plating in media exposed to oxygen. We decided to test acid survival under conditions in which culture growth, acid exposure, dilution, and plating were conducted in a chamber excluding oxygen (dissolved oxygen concentrations below 6 µM).

Under aeration, acid survival requires the glutamate-dependent acid response (*gad*) system and the sigma factor σ^S^ subunit of RNA polymerase (*rpoS*) [4], [8]. In the Gad system, glutamate decarboxylase consumes a proton from the bacterial cytoplasm to convert glutamate into γ-butyric acid (GABA) and carbon dioxide. GABA is exported to the periplasm by the antiporter in exchange for new glutamate [12], [13]. The net consumption of protons raises the cytoplasmic pH to a level that maintains viability [4]. Other factors contributing to acid stress response include the arginine and lysine decarboxylases [14],[15] as well as up-regulation of cyclopropane fatty acids (Cfa) which modifies membrane phospholipids so as to enhance acid resistance [16].

In the human gastrointestinal tract, enteric bacteria experience variable oxygen levels. The rectal region maintains a fairly stable range of oxygen concentration at or below 3 µM O_2_ (1.5% saturation) [17]. The stomach, however, undergoes transient fluctuations in O_2_ concentration as well as low pH, owing to the periodic input of oxygenated food. Despite intermittent increases in O_2_ levels, the gastric epithelium harbors obligate anaerobes such as *Clostridium* and *Veillonella* species, as well as many facultative anaerobes [18], [19]. *Helicobacter pylori*, which primarily occupies the lower stomach gastric lining, grows optimally in a microaerobic environment (6–15 µM O_2_) [20]. Exclusion of oxygen has been proposed to enhance acid survival, because anaerobic growth increases expression of acid stress mechanisms such as lysine and arginine decarboxylases [21].

Much of the *E. coli* response to decreasing O_2_ concentrations is mediated by the FNR regulon. When dissolved oxygen levels fall below 10 µM, FNR monomers begin to dimerize as the iron-sulfur centers oxidize [22], [23], and the cell's metabolism transitions to anaerobiosis [24], [25]. FNR-induced genes encode alternative terminal electron acceptors, hydrogenase maturation proteins, periplasmic chaperones, and functional replacement proteins for components of aerobic metabolism. Aerobic genes, including those providing protection from reactive oxygen species (ROS), are down-regulated.

ROS stress is an important factor for the acid stress response under anoxic conditions [26]. Anaerobic growth at low pH up-regulates ROS stress genes, suggesting that low pH amplifies ROS stress [9], [10]. One source of oxidative stress under laboratory conditions is the Maillard reaction, which occurs in broth medium during autoclaving [27]. In the Maillard reaction, amino acids react with sugar to produce ketosamines and other potentially toxic products, as well as hydrogen peroxide [28]. In well aerated cultures, hydrogen peroxide is eliminated by catalases including KatE, KatG and AhpC [29], [30] but the effects of other Maillard reaction products are uncertain. Under low oxygen, catalases are down-regulated by anaerobic regulators such as FNR.

In this report, we excluded oxygen during the entire extreme-acid experiment (overnight culture, extreme-acid exposure, dilution, and plating), using a controlled atmosphere chamber maintained at <6 µM O_2_. We found that the major genes required for acid resistance under aeration are not required when oxygen is excluded. We also revealed a role for autoclave-generated toxic products in acid resistance.

## Methods

### Bacterial strains and growth

*E. coli* K-12 derivative W3110 [31] was used as the background for all mutant strains. Gene deletion alleles with kanamycin resistance cassettes were transduced from Keio collection strains into W3110 via P1 phage transduction [32]. Bacteria were cultured on Luria Bertani agar with 7.45 g/l potassium chloride (LBK) and 50 µg/ml kanamycin. Single gene knockout mutant strains included: JLS0807 (W3110 *gadC*), JLS9405 (W3110 *rpoS*), JLS1034 (W3110 *cfa*), JLS0925 (W3110 *hypF*), and JLS1115 (W3110 *fnr*). Bacterial strain freezer stocks were sampled no more than 5 times, to avoid loss of acid resistance associated with thawing and refreezing.

### Acid survival assays

The conditions for testing acid resistance (survival in extreme acid) were based on those described previously [11] with modifications. Cultures were grown overnight in LBK buffered with 100 mM 2-(*N*-morpholino)ethanesulfonic acid (MES) at pH 5.5 to up-regulate acid response systems [1]. Cultures were exposed to extreme acid (LBK pH 1.2–2.0) for 2 h in a 1∶200 (aerated) or 1∶400 (oxygen exclusion) dilution, and then were serially diluted in M63 minimal media (pH 7.0) to a final dilution of 1∶400,000 (aerated) or 1∶80,000 (oxygen exclusion). 50 µL of the final dilutions were spread onto agar plates. Colonies from these dilutions were grown up at 37°C then counted and log transformed. A control was completed in the same manner as for acid exposure. Cells from the overnight cultures were diluted in M63 minimal media pH 7.0. The final dilution of control cells was the same as that of pH 2.0 exposure under both aerated and oxygen exclusion conditions. Colony counts for each replicate were log transformed and a log ratio of average log values from the replicates of each condition from pH 2 to pH 7 was used to calculate percent survival. The standard error of the mean (SEM) was calculated from the log ratios of daily replicates (n = 5 or 6). Two-tailed, unpaired heteroscedastic t-Tests were completed on each strain to compare the effects of different strains or exposure conditions.

### Oxygen exclusion

Oxygen was excluded by use of a controlled atmosphere chamber (Plas Labs). External atmosphere was initially purged from the chamber 9 times with a vacuum pump. Following each purge, a gas mixture of 5% CO_2_, 10% H_2_, and 85% N_2_ was introduced to restore neutral pressure. Remaining O_2_ was catalytically removed by a palladium canister affixed atop a heating unit that maintained temperature at 37°C. Liquid media and materials to be used were placed in the chamber for at least 18 hours before use; agar plates were introduced at least 4 hours before use. Dissolved oxygen concentration was measured using an Oakton Hand-held Dissolved Oxygen Meter (DO110) with the electrode immersed in distilled water. The oxygen level in the chamber was maintained below <6 µM.

### Quantitative reverse transcriptase polymerase chain reaction (qRT-PCR)

qRT-PCR based on the method of Refs. [9] and [15]. *E. coli* K-12 W3110 and JLS1115 (W3110 *fnr*) were cultured in the controlled atmosphere chamber with LBK buffered with 100 mM MES at pH 5.5. Bacterial RNA was stabilized by rapid addition of an ice-cold solution of 10% phenol in ethanol, a procedure that avoids induction of acid-stress genes. The RNA was then purified using the RNeasy Kit (Qiagen) followed by DNase treatment (Ambion). Targeted primer sequences were designed using Primer Express (Applied Biosystems) and supplied by Invitrogen. The SYBR Green PCR One-Step Protocol was used so that that reverse transcription of RNA and the amplification of transcripts took place simultaneously (Applied Biosystems). Reactants included: 0.1 nM forward primer, 0.1 nM reverse primer, and 50 ng of target RNA, 52% SYBR Green (v/v). Cycling conditions were: reverse transcription for 30 min at 48 °C and 10 min at 95 °C, 40 cycles of 15 s denaturation at 92°C, and extension for 1 min at 60 °C. Gene expression was normalized to the total RNA in each reaction, in order to avoid dependence on “housekeeping” genes that are depressed by acid [9]. For each gene, the average cycle time (C_t_) value was determined from three biological replicates run in triplicate. No-template and no-reverse transcriptase controls were performed for each gene.

## Results

### Extreme-acid survival without oxygen

**Figure 1:**
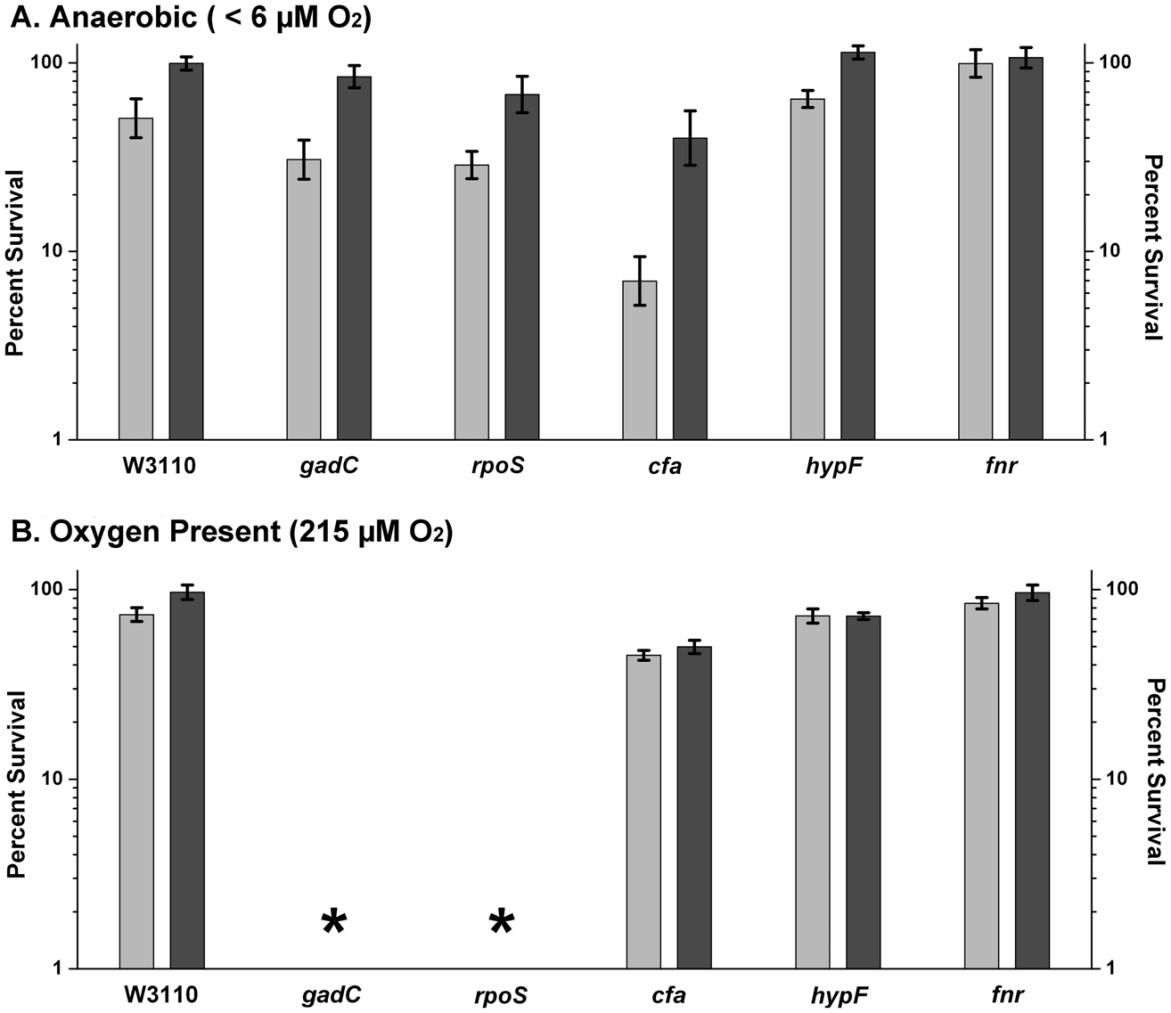
Acid survival of mutant strains. Single-gene mutants of *E. coli* K-12 strain W3110 were constructed as described under Methods. Strains defective for *gadC*, *rpoS*, *cfa*, *hypF*, and *fnr* were cultured overnight and exposed to pH 2.0 for 2 hours before being diluted 1∶80,000 and 1∶400,000 under anoxic and aerated conditions, respectively. Dilutions were then plated allowing colonies to grow up overnight at 37° C. The number of colonies per plate was log transformed and a ratio of acidic exposure – control (pH 7.0) and a percentage was calculated from that ratio. Extreme acid medium was autoclave sterilized (Light bars); or filter sterilized (dark bars). Error bars indicate SEM (n = 5 or 6). * denotes undetectable colony counts on dilution plates and a corresponding survival of <1%.

Under aeration (215±5 µM O_2_), the *gad* and *rpoS* regulons are required for acid survival [1]. We observed the survival of *rpoS* (JLS9405) and *gadC* (JLS0807) deletion mutants in extreme acid cultured with aeration or in the chamber, where oxygen levels were measured at less than 6 µM (Fig. 1). Under aeration, *rpoS* and *gadC* strains showed less than 1% survival after exposure for 2 hours in LBK pH 2.0. Anaerobic cultures of the same strains survived at pH 2.0 at levels comparable to those of the parent strain. Anaerobic cultures of W3110 strains also survived 50–90% in M63 minimal medium pH 2.5, with or without 1.5 mM glutamate (data not shown). Thus, glutamate and the Gad regulon were not required for extreme-acid resistance under oxygen exclusion; nor was RpoS, which induces Gad expression.

Cyclopropane fatty acid biosynthesis has a reported role in acid resistance [16], [33]. In our experiments, *cfa* deletion mutants showed nearly complete survival under aeration and showed no inhibition by autoclaved or filtered exposure media (t-test, p-values >0.2, n = 4). Under oxygen exclusion, the *cfa* strain had a significantly lower survival rate in autoclaved medium (<10% survival, p-value <0.05, n = 5), though in filtered medium the survival percentage was within the range considered acid resistant (>10%). A *hypF* deletion strain showed no loss of survival in extreme acid (Fig. 1). HypF is required for maturation of all the *E. coli* hydrogenase complexes (Hya, Hyb, and Hyc) [9], [34], [35]. Thus, none of the *E. coli* hydrogenases were essential for anaerobic extreme-acid survival.

In Fig. 1, all strains exposed at pH 2 in autoclaved medium showed significantly lower survival than in filter-sterilized medium when exposed under oxygen exclusion, with the exception of the *fnr* deletion strain. Differences between autoclaved and filtered exposure media were determined to be statistically significant if their t-test yielded p-values <0.05 (Table 1). With aeration the difference between autoclaved versus filtered medium at pH 2 was small or insignificant.

**Table 1:** Statistical analysis of survival assays for Figure 1.

Anaerobic		Aerated	
Strain	p-value	Strain	p-value
W3110	0.040	W3110	0.002
*gadC*	0.009	*gadC*	N/A
*rpoS*	0.009	*rpoS*	N/A
*cfa*	0.003	*cfa*	0.194
*hypF*	0.005	*hypF*	0.981
*fnr*	0.620	*fnr*	0.278

**Figure 2:**
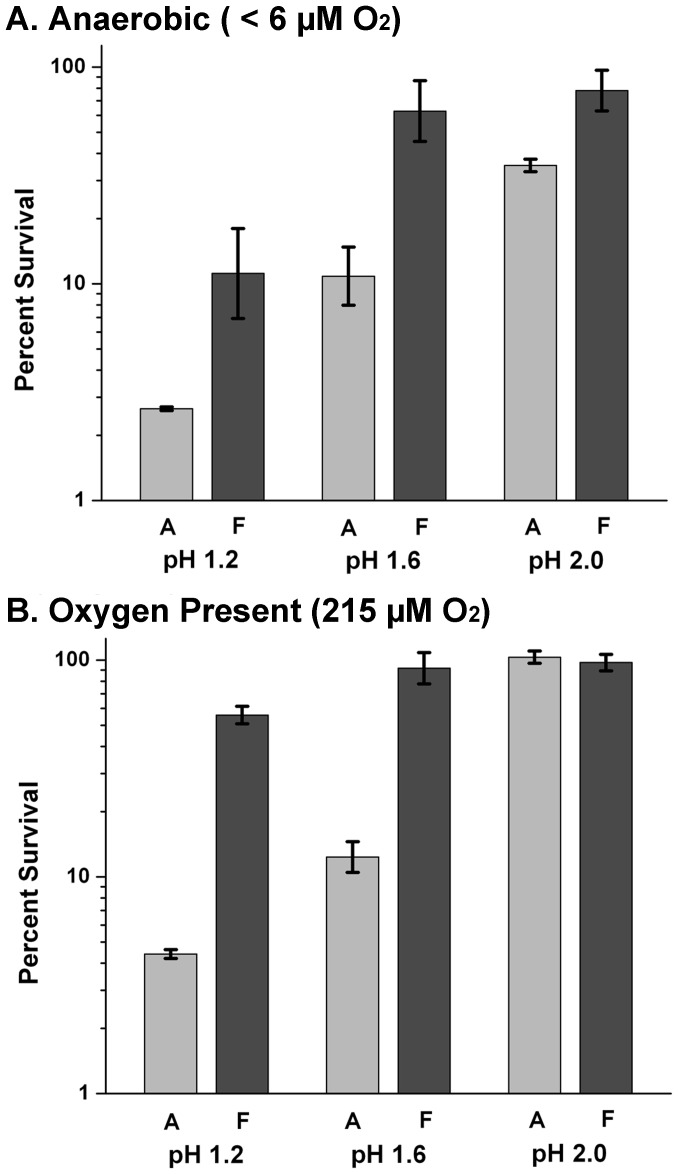
*E. coli* survives at pH lower than pH 2.0. Overnight cultures of *E. coli* K-12 strain W3110 were grown in LBK 100 mM MES pH 5.0. These cultures were exposed to medium at pH 1.2; 1.6;and 2.0, respectively, for two hours. Dilutions from exposed cells were completed as in **Fig. 1**. Strains were exposed in autoclave-sterilized medium (light bars) and in filtered medium (dark bars). Error bars indicate SEM (n = 5 or 6).

The effect of autoclaving on acid survival was tested further at pH values below 2.0, comparing acid exposure with aeration versus oxygen exclusion (Fig. 2). In anaerobic filtered media at pH 1.6 and 2.0, a small difference in acid survival was seen (65% and 75% respectively). Aerated cultures survived above 55% in filtered media at pH 1.2, 1.6, and 2.0. At pH values lower than pH 2.0, autoclaved medium showed lower *E. coli* survival than filtered medium. Overall, at pH 1.6 or 1.2, both aerated and anaerobic cultures showed decreased survival in autoclaved medium.

**Figure 3:**
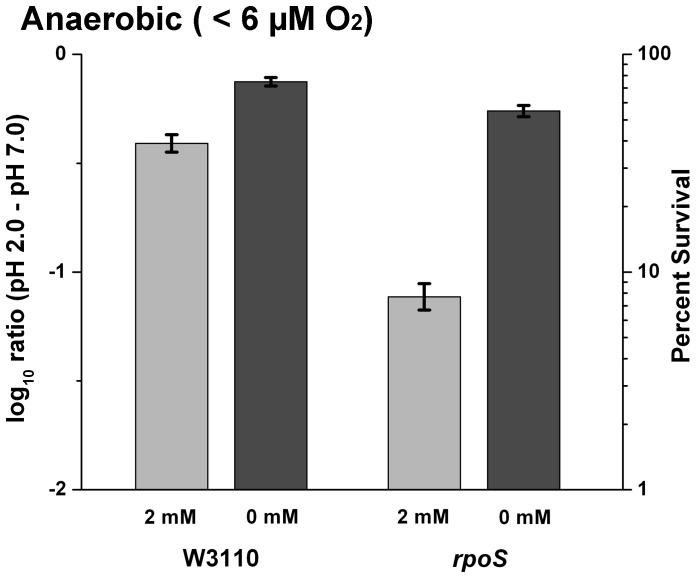
*rpoS* mutant survival is affected by low concentrations of H_2_O_2_. Overnight cultures of W3110 and our *rpoS* mutant were exposed to filter sterilized LBK pH 2.0, with and without 2 mM H_2_O_2_ under both oxygen exclusion and aeration and were serially diluted to final dilutions of 1∶80,000 and 1∶400,000, respectively. Error bars indicate SEM (n = 5 or 6).

We hypothesized that the sensitivity to autoclaved medium at pH 2 was due to the production of H_2_O_2_ during the Maillard reaction. To test this possibility, we repeated our extreme-acid survival assays in filtered medium with added H_2_O_2_. In the anaerobic chamber, 2 mM H_2_O_2_ had a small effect on extreme-acid survival of strain W3110, and decreased extreme-acid survival of *rpoS* to below 10% (Fig. 3). Thus, *rpoS* strain showed greater sensitivity to H_2_O_2_ than did the parental strain (t-test, p-value <0.01), although the effect of autoclaved medium showed no significant difference between the two strains.

### Deletion of *fnr* eliminates sensitivity to autoclaved medium

**Figure 4:**
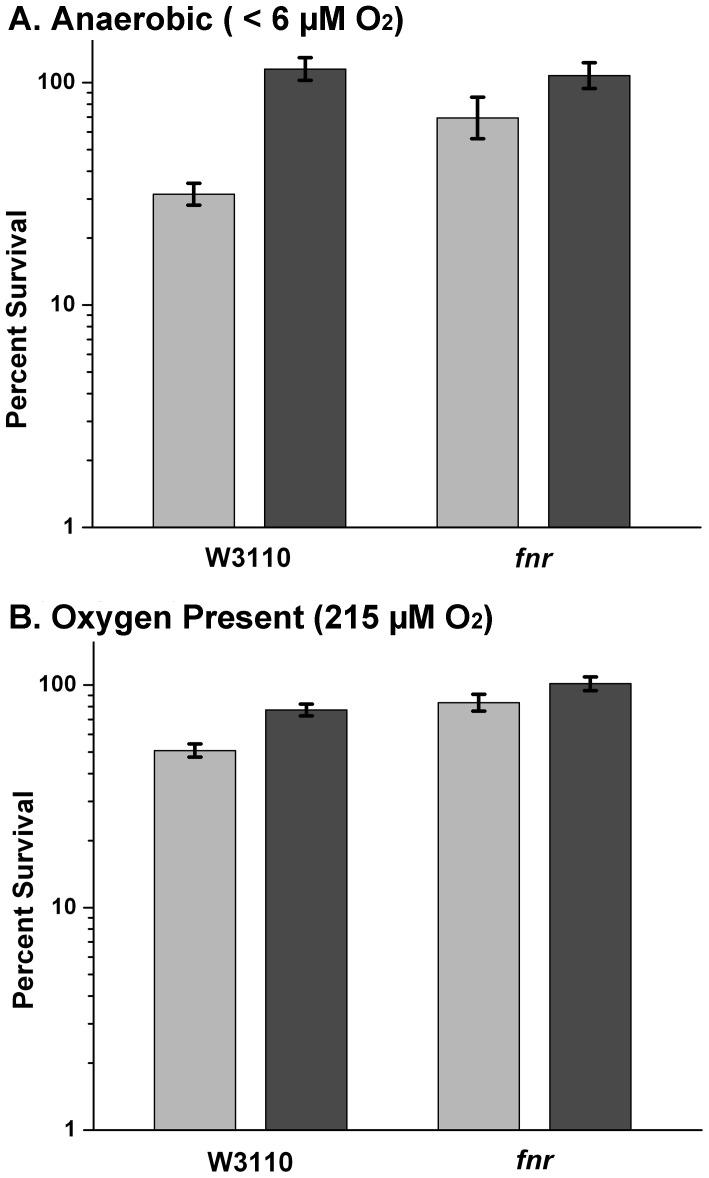
FNR deletion enhances anaerobic acid resistance in autoclaved medium. Cultures of W3110 and JLS1115 (W3110 *fnr*) were grown in LBK buffered with 100 mM MES at pH 5.5. Bacterial cultures were exposed to LBK pH 2.0 for 2 hours. Exposure tubes were serially diluted 1∶400,000 and 1∶80,000 aerobically and anaerobically, respectively, and plated. Viable colonies on each of 6 replicate plates were counted and log transformed. The log ratios (pH 2.0– pH 7.0) were then used to calculate the percentage of cells surviving in comparison to a pH 7.0 exposure control. The survival response of W3110 and JLS1115 were compared in autoclaved medium (light bars) and filtered medium (dark bars). Error bars indicate SEM (n = 5 or 6).

The *fnr* deletion strain showed little or no difference between extreme-acid survival in autoclaved versus filtered medium (Fig. 1). The enhanced acid resistance with the *fnr* strain was confirmed in experiments pairing the mutant with the parent strain W3110, using freshly autoclaved medium in which volatile components would be maximally retained. A representative experiment is shown in Fig. 4, in which autoclave-product sensitivity appeared only for W3110 exposed at pH 2. The effect of autoclaved medium was greater under oxygen exclusion (<6 µM O_2_).

**Figure 5:**
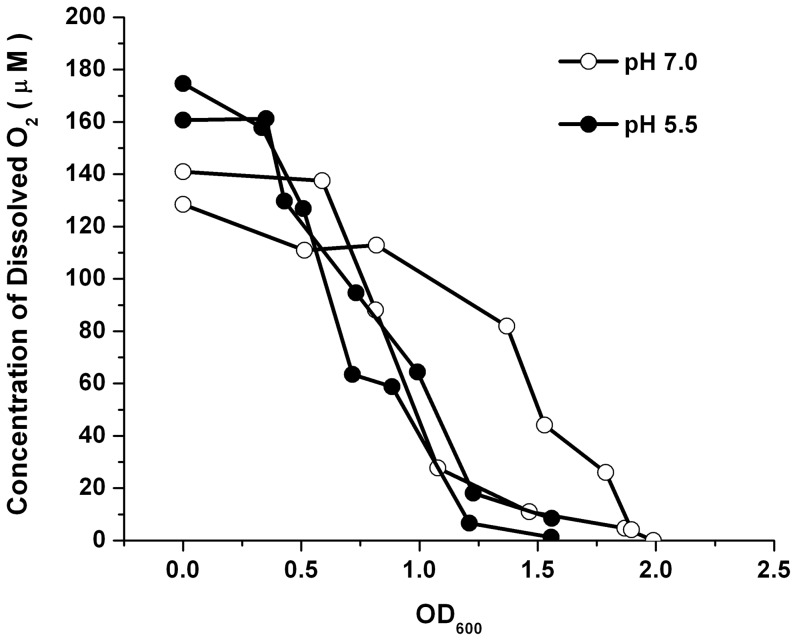
Oxygen levels drop below levels of detection by OD_600_ = 1.5. Overnight cultures were diluted 1∶200 into 100 ml of LBK at pH 7.0 (open circles) and pH 5.5 (solid circles). Cultures were incubated in a 37°C water bath rotating at 160 rpm in 250-ml baffled flasks. Optical density (λ = 600) and dissolved oxygen levels were recorded every 20 min after the first hour of incubation. Dissolved oxygen concentrations were plotted as a function of OD_600_.

Because FNR is activated only below 10 µM O_2_ [22], [23], we measured the oxygen levels in our aerated cultures in order to assess the possibility that FNR-dependent expression occurs. The actual availability of oxygen in the cytoplasm depends upon the O_2_ concentration in the solution, the diffusion rate into the cell, and the rate of consumption by metabolism. While diffusion does not generally limit oxygen availability, oxygen consumption is a major limiting factor for growing cells, even during vigorous aeration [36]. We measured dissolved oxygen concentrations under conditions of exponential growth in baffled flasks rotated at 160 rpm at 37°C (Fig. 5). An overnight culture of *E. coli* W3110 was diluted 200-fold into fresh buffered LBK. The initial oxygen concentration range was between 130–180 µM, already somewhat less than that of air-saturated distilled water (215 µM). As the bacteria grew, the dissolved oxygen level declined steadily to 10 µM as the culture reached OD_600_ values of between 1.2–1.8, and ultimately fell below 3 µM (the lower limit of detection by our meter). The decline of oxygen as a function of culture density was similar for cultures buffered at pH 7.0 or at pH 5.5, the pH at which bacteria were cultured to induce genes for extreme-acid survival. Thus, it is likely that all our aerated cultures showed some FNR-dependent gene expression as they entered stationary phase.

**Figure 6:**
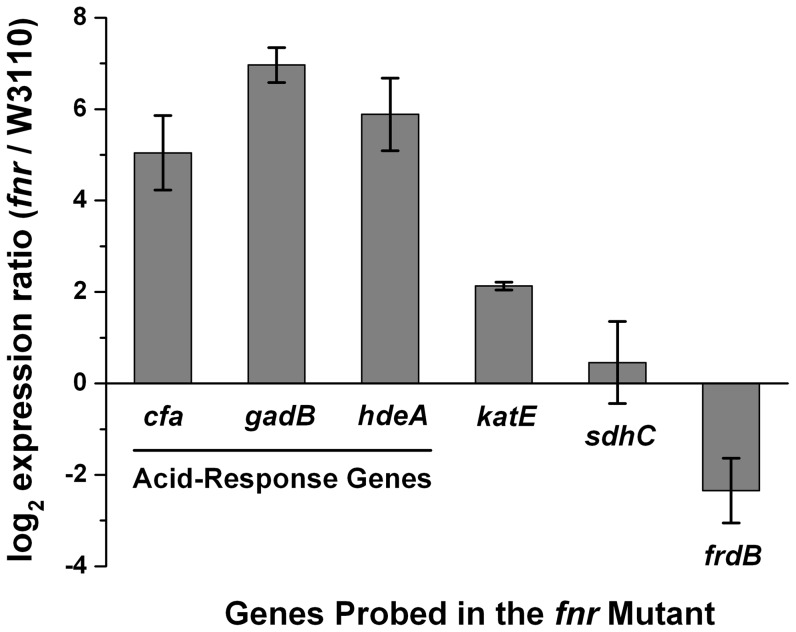
Gene expression affected by *fnr* during growth at pH 5.5. RNA was isolated from anaerobic cultures of JLS1115 (W3110 *fnr*) (gray bars) grown to stationary phase at pH 5.5 in buffered LBK. qRT-PCR was used to measure the differential expression of mRNA levels for *cfa*, *gadB*, *hdeA katE*, *sdhC*, and *frdB* in the *fnr* mutant compared to the wild-type using primers listed in Table 2. Positive values denote higher expression in the *fnr* mutant than in the wild-type and vice-versa. Error bars represent SEM, n = 3 (RNA from independent cultures). The expression profile for each gene was verified in triplicate.

Some of the genes down-regulated by FNR for anaerobic metabolism may actually enhance survival in extreme acid in the presence of H_2_O_2_ or other substances generated during autoclaving [25], [37]–[39]. We investigated whether the absence of FNR might relieve its repression of genes known to contribute to acid resistance (Fig. 6). RT-PCR was performed on W3110 and JLS1115 (W3110 *fnr*) cultured excluding oxygen at pH 5.5, conditions typical of those for the extreme-acid test. Known aerobic acid-resistance genes (*cfa*, *gadB*, *hdeA*) showed up-regulation in the *fnr* strain. *katE* was up-regulated 2-fold in the *fnr* mutant. The *sdhC* (succinate dehydrogenase) and *frdB* (fumarate reductase) are shown for comparison; these genes are not repressed by Fnr, and are not known to contribute to acid resistance but served as null and negative controls, respectively. These observations of FNR-mediated differential expression are consistent with previous reports of FNR regulation in cultures grown at pH 7 [24].

**Table 2:** Primers used for qRT-PCR.

Primer	Sequence
*cfa*-forward	GAGAACCAACTCCCCCATCA
*cfa*-reverse	ACGAGCGCCGGCAAT
*frdB*-forward	CGGCGTATGGAGCTGTACTTT
*frdB*-reverse	GACGTGTTTCGGGCAGACTT
*gadB*-forward	GCGGATGGCGATGAA
*gadB*-reverse	GTTTGCCTGCAGCTTCCATAC
*hdeA*-forward	TCCTGGCTGTGGACGAATC
*hdeA*-reverse	AGCGCTTCAGCAAAACCAA
*katE*-forward	GCGGCGGTTTTGAATCATAC
*katE*-reverse	CGCTCGCGAACTTTATTGC
*sdhC*-forward	CGGCGATAGCGTCCATTCT
*sdhC*-reverse	CCACTGCAACAAAGGTGATCA

## Discussion

We show that when oxygen is excluded from *E. coli* cultures, key genes for aerobic extreme-acid survival are not required. The lack of effect of *rpoS* deletion was particularly remarkable, as RpoS is considered essential for both acid and base resistance [1], [3]. Even hydrogenase 3 was not required for acid survival without oxygen, although hydrogenase enhances acid resistance of anaerobic overnight cultures exposed to acid in semi-aerobic media [11]. The *cfa* mutant significantly impacted anaerobic acid resistance, especially in autoclaved medium. Increased production of cyclopropane fatty acids protects *E. coli* from acid [33], [40].

The above findings show that simple categories of “aerobic” versus “anaerobic” are insufficient to describe the actual states of oxygen availability for enteric bacteria. Oxygen is available over a continuum of concentration, on a log scale analogous to that of pH. We might define empirical ranges as follows:

- **Aerobic (130–215 µM)** Log-phase cultures, with fully expressed aerobic metabolism
- **Semi-aerobic (10–130 µM)** Late log phase to early stationary phase
- **Anaerobic transition (1–10 µM)** Stationary phase, progressive activation of FNR
- **Anoxic (<1 µM)** Oxygen unavailable

Such a scale helps keep different bacterial environments in perspective. For example, the human gut epithelium is often described as lacking oxygen although its oxygen levels fall within the range of anaerobic transition, where some oxygen remains available to gut bacteria.

Because *E. coli* is capable of nearly complete survival under both aerated and oxygen-excluded exposure at pH 2.0, it was of interest to study its survival capabilities in media at even lower levels of pH. In aerated filtered medium, *E. coli* survived above 90% at pH 1.6 and survived over 50% at pH 1.2. Under oxygen exclusion, survival at pH 2 was only slightly inhibited at pH 1.6 (range of 65–90%), and partly inhibited at pH 1.2 (just above 10%; Fig. 2). Survival of enteric bacteria at such a low pH is remarkable, given the need to maintain cytoplasmic pH at a minimum of pH 4.8 for viability [1].

Below pH 2, both aerated and anaerobic cultures showed a substantial loss of survival in autoclaved medium. Similar differences in survival between autoclaved and filtered broth media were observed at pH 2 in the various mutant strains (Fig. 1). H_2_O_2_ generated during autoclaving is known to inhibit *E. coli* as well as marine bacteria and unculturable organisms [28], [41]–[43]. However, the H_2_O_2_-sensitive *rpoS* mutant survived in autoclaved medium at levels lower than those of the parental strain; thus, it is likely that products other than H_2_O_2_ mediate the effect of autoclaved medium on extreme-acid survival. Our findings suggest that in general, in studies using autoclaved medium, the ability of enteric bacteria to survive acid may be underestimated.

The effect of autoclaved medium was not observed in the *fnr* mutant (Fig. 1, Fig. 4). Possible acid resistance factors up-regulated in the *fnr* mutant include KatE, Cfa, the Gad regulon, and the periplasmic chaperone protein HdeA. It may be that enhanced expression of several acid resistance factors together increases survival of the *fnr* deletion strain.

The increased anaerobic acid resistance in the *fnr* strain is of interest as an example of how loss of regulation can enhance fitness under an altered stress condition. Similar loss of regulators appears in evolution of antibiotic persisters, cells that survive antibiotic exposure in a dormant state [44], [45]. Further investigation of regulator loss may provide addition clues to mechanisms of extreme-acid resistance in enteric bacteria.
